# Exosomal miRNA-139 in cancer-associated fibroblasts inhibits gastric cancer progression by repressing MMP11 expression

**DOI:** 10.7150/ijbs.33750

**Published:** 2019-08-22

**Authors:** Guifang Xu, Bin Zhang, Jiahui Ye, Shouli Cao, Jiajun Shi, Yan Zhao, Yongsheng Wang, Jianfeng Sang, Yongzhong Yao, Wenxian Guan, Jinqiu Tao, Min Feng, Weijie Zhang

**Affiliations:** 1 Department of Gastroenterology, Affiliated Drum Tower Hospital of Nanjing University Medical School, Nanjing, Jiangsu Province, China;; 2 Department of General surgery, Affiliated Drum Tower Hospital of Nanjing University Medical School, Nanjing, Jiangsu Province, China;; 3 Department of Respiratory Medicine, Affiliated Drum Tower Hospital of Nanjing University Medical School, Nanjing, Jiangsu Province, China.

**Keywords:** Gastric cancer, CAFs, exosome, MMP11, miR-139, cancer progression

## Abstract

Solid tumors consist of various types of stromal cells in addition to cancer cells. Cancer-associated fibroblasts (CAFs) are a major component of the tumor stroma and play an essential role in tumor progression and metastasis in a variety of malignancies, including gastric cancer. However, the effects of CAFs on gastric cancer cells' progression and metastasis are not well studied. Here we show that matrix metalloproteinase 11 (MMP11) in exosomes secreted from CAFs can be delivered into gastric cancer cells. Gastric CAFs promote gastric cancer cell migration partially through exosomal MMP11. Moreover, MMP11 is overexpressed in exosomes purified from plasma of gastric cancer patients and tumor tissues and associated with overall survival of gastric patients. We also find that MMP11 is negatively regulated by exosomal miR-139 in the CAFs of gastric cancer. Exosomal miR-139 inhibits tumor growth and metastasis of gastric cancer cells by decreasing the expression of MMP11* in vitro* and *in vivo*. Thus, we propose that exosomal miR-139 derived from gastric CAFs could inhibit the progression and metastasis of gastric cancer by decreasing MMP11 in tumor microenvironment.

## Introduction

Gastric cancer (GC) is one of the most aggressive malignant tumors with unfavorable prognosis, and the incidence and mortality of GC is increasing in China. It was documented that 679,100 new cases diagnosed with GC in 2015, which account for 15.8% of the total number of newly diagnosed cancer cases, and as a result, 498,000 deaths occurred 2. Despite advances in the surgical techniques and therapeutic strategies, the outcome of GC patients was still dismal due to tumor relapse and metastasis 2-4. However, the exact mechanisms of GC recurrence and metastasis remain unclarified.

Accumulating studies have demonstrated that the cellular cross-talk between cancer cells and surrounding stromal in tumor microenvironment (TME) play a vital role in regulating cancer progression and therapeutic resistance^3^-^6^. As an indispensable component of TME, cancer-associated fibroblasts (CAFs) are considered as an essential part that produce various tumor components, growth factors and chemokines 3. Despite the availability of numerous researches describing the vital contribution of CAFs on oncogenesis in multiple types of solid tumor, a detailed mechanism of the biology of cancer-stroma interaction in GC remains obscure and is largely under investigated.

Exosomes are nano-sized microvesicles composed of lipid bilayer and contain various bioactive molecules, including miRNA, long non-coding RNA, circular RNAs, which are considered as mediators for intercellular communication 7,8. Recently, exosomal microRNAs (exomiRs) have drawn much attention because numerous studies have demonstrated that exomiRs can influence many hallmarks of cancer, such as cancer development, progression, and metastasis 9-12, and more evidences have suggested that exomiRs are highly suitable candidates for use as biomarkers in an era of precision medicine 13,14. However, the mechanisms of exomiRs in regulating the dynamic crosstalk among cancer cells and CAFs and shape the TME are still elusive.

In this study, we identify that gastric CAFs promote gastric cancer cell migration partially through exosomal MMP11. We also find that MMP11 is negatively regulated by exosomal miR-139 in the CAFs of gastric cancer. Exosomal miR-139 inhibits tumor growth and metastasis of gastric cancer cells by decreasing the expression of MMP11 *in vitro* and *in vivo*. Of clinical significance, MMP11 is overexpressed in exosomes purified from plasma of gastric cancer patients and tumor tissues, and associated with overall survival of gastric cancer patients. Our findings indicate that exosomal miR-139 derived from gastric CAFs could inhibit the progression and metastasis of gastric cancer by targeting MMP11 in TME.

## Materials and methods

### Human blood samples and tumor samples

Human plasma and tumor samples of GC patients and healthy controls were obtained from patients or donors who were admitted to Nanjing Drum Tower Hospital, Medical School of Nanjing University from January 2013 to January 2016. All GC patients were confirmed histologically or pathologically in clinical practice by two independent pathologists. The pathological type of each cancer was determined to be adenocarcinoma. Written consent was obtained from all participating patients and healthy controls. The Ethics Committee of Nanjing Drum Tower Hospital approved this research. Plasma and tumor samples were prepared and stored at -80℃ for use.

### Isolation of exosomes from cultured supernatants and plasma for characterization

Exosomes, as protocol instructed (Thermo Fisher Scientific, USA), were obtained from cell cultured supernatants and plasma by exosome isolation kit. Resuspend exosome pellet in 1/10 to 1/100 of original volume using sterile phosphate buffered saline (PBS) and aliquot in cryogenic vials and store at -80℃ until usage. After separation, exosomes were fixed in 0.01 M phosphate buffer solution (PBS, containing 4% paraformaldehyde, PH 7.4) at 4ºC overnight. On the next day, after being washed with PBS, the exosomes were fixed in 1% OsO4 for 30 min. After being rinsed with distilled water, the exosome precipitate was dehydrated in concentration gradient alcohol, and stained by 1% uranyl acetate for half an hour, then embedded in TaAb 812. After polymerization at 60 ºC overnight, the precipitate was sliced, and the ultra-thin sections were observed under a transmission electron microscope.

### Immunohistochemical staining

Briefly, after antigen retrieval, tissue sections were incubated with a rabbit polyclonal anti-α-SMA-antibody (1:200 dilution; Proteintech, Chicago, IL, USA) overnight at 4°C, followed by incubation with a biotinylated secondary antibody and an avidin-biotin peroxidase complex (ZSGB-Bio, Beijing, China). Then, the immune reactions were developed by adding DAB chromogen substrate solution (ZSGB-Bio, Beijing, China) to the slides. Harris hematoxylin was used for counterstaining. Negative controls were run in parallel with all reactions. All specimens were scored by a board-certified pathologist. The immunohistochemical staining scoring for α-SMA was evaluated as “low” or “high” expression, regarding the rate of positive cells for each sample as described previously 15.

### Colony formation assay

The cells were measured via colony formation assays following exposure to ionizing radiation (IR). Cells were treated with various doses of radiation after being seeded in 6-well plates at different densities (200, 400, 600 or 1,000 cells/well with 0, 2, 4, 6 or 8 Gy of radiation). Then, the cells were incubated for another 12 days until colonies appeared. After being fixed with carbinol for 25 min and stained with Giemsa stain (Applichem, Germany) for 35 min, the colonies (each colony with at least 50 cells) were counted. Experiments were performed in triplicate. GraphPad Prism 5.0 software was used to fit the data to a multi-target, single-hit model; SF (survival fraction) = 1 - (1 - e-D/ D0 )N, where D0 (mean lethal dose) is the single dose of radiation that can kill 63% of the cells and N represents the number of intracellular radiation-sensitive areas.

### Transwell migration and invasion assay

Transwell migration and invasion assay were performed using transwell chambers without (migration) or with (invasion) Matrigel (BD Sciences, Sparks, MD, USA). The N87 and AGS cells were seeding in the six well plates and transfected with pcDNA3.1- FOXF1-AS1 or pcDNA3.1 control plasmid using Lipofectamine 2000 (Invitrogen, Carlsbad, CA, USA) according to the manufacturer's instructions. The stable clones were selected by adding G418 and the expression of FOXF1 was validated by qRT-PCR. The stable transfected N87-FOXF1-AS1 and AGS-FOXF1-AS1 and control cells at an approximate density of 1 × 10^5^ were suspended and seeded in the upper chambers of 24 well plates with FBS free medium. After 18 h, cells that migrated were stained by 0.5 % crystal violet solution for 15 min and counted. For invasion assays, transwell membrane was prepared with matrigel for plating infected cells. After 24 h, cells that migrated were stained by 0.5 % crystal violet solution for 15 min and counted.

### qRT-PCR analysis

Total RNA was isolated from exosomes and tissues using a miRNeasy mini kit (Qiagen, Hilden, Germany) and from plasma using the exoRNeasy Serum/Plasma Midi Kit (Qiagen, Hilden, Germany). The quantity of mature miRNAs expressed was determined by quantitative real-time PCR (qPCR) using a miScript II RT kit (Qiagen, Hilden, Germany) and miScript SYBR Green PCR Kit (Qiagen, Hilden, Germany) with Qiagen predesigned primers. All kits were used according to the manufacturer's instructions. A U6 transcript was used as an internal control to normalize RNA input. Analysis and fold change were determined using the comparative threshold cycle (Ct) method 16.

### Western blot

The concentrations of total proteins which were isolated from cells were detected by the BCA Assay Kit (Thermo Fisher Scientific, USA). Then proteins were separated by 10% SDS-PAGE and transferred onto PVDF membranes (Millipore, USA). 5% non-fat milk was used to block the membranes for 2 hours, which were incubated overnight at 4°C with the primary antibody, including CD63 and CD9 and SFRP1 (Sigma, USA, 1:1000) and GAPDH (Santa Cruz Biotechnology, USA, 1:1000). GAPDH was used as an internal control.

### Xenograft models

Female nude mice (BALB/c-nu, 6-8 weeks) were housed in a pathogen free animal facility with access to water and food and allowed to eat and drink. All experimental procedures were performed in accordance with protocols approved by the Institutional Animal Care and Research Advisory Committee. The N87-H and N87-L cells were transfected by pLenti-luciferase virus. The N87-L-Exosomes cells were established by incubation N87-L cells with the purified Exosomes from N87-H cells. After a left-side upper abdominal incision was made, the stomach of the nude mouse was gently exteriorized. Mice were injected with 2 × 10^6^ of N87-H, N87-L and N87-L-Exosomes cells in the gastric of mice. Seven days after the injection of tumor cells, tumor primary site or metastasis site was observed by bioluminescent imaging.

### Luciferase report system

The 3'-UTR sequence of MMP11 was amplified from normal genomic DNA of human and then subcloned into the luciferase reporter vector. N87-L cells (5 × 10^4^) were seeded in 24-well plates and co-transfected with wild-type (wt) or mutant (mut) 3′-UTR reporter plasmids and P-miR-196a-1 or P-miR-control using Lipofectamine 3000. After 48 h, cells were tested for luciferase activity using the Dual-Luciferase Reporter Assay System (Promega) according to the manufacturer's instructions. The experiments were performed in triplicates.

### Statistical analysis

SPSS 22.0 and GraphPad Prism 5 statistical software were applied on the experimental data analysis. We select Wilcoxon rank sum test for two independent and paired samples to take the test, and select Mann-Whitney U test for variable analysis. P<0.05 was considered as statistically significant difference.

## Results

### Increased CAFs promoted gastric cancer cell migration

**Figure 1:**
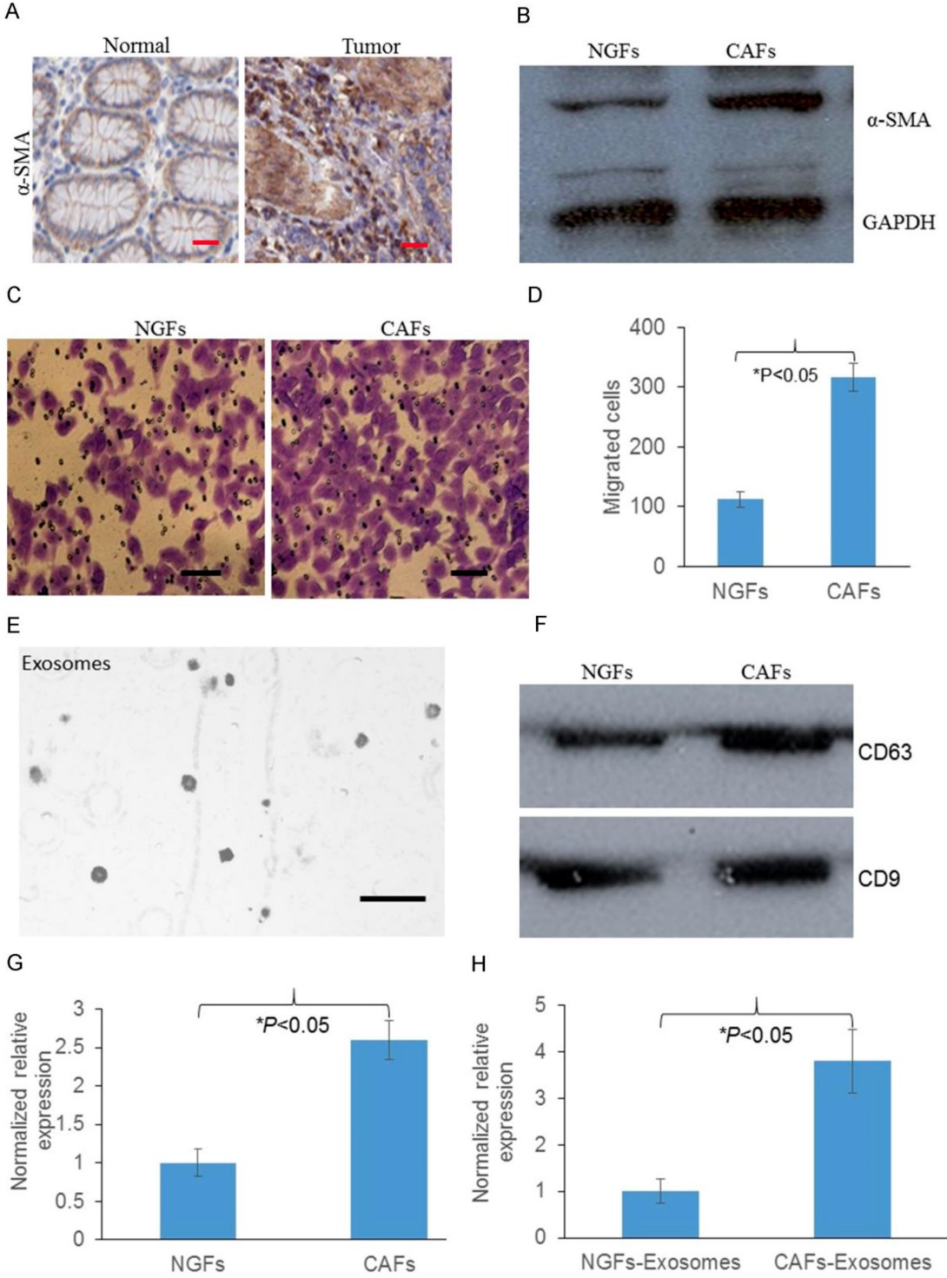
Overexpression of MMP11 in exosomes of gastric CAFs contributes to migration.

To detect whether fibroblasts were increased in tumors, we first analyzed the expression of α-SMA, which is indicated as the biomarker for fibroblast expression. We found that protein expression of α-SMA was significantly increased in GC tumors compared with normal tissues, indicating GC tumors contain more activated fibroblasts (Fig. 1A). Then we asked whether CAFs contain more activated fibroblasts, we analyzed the expression of α-SMA in normal gastric fibroblast cells (NGFs) and CAFs. As Fig 1. B showed that compared with NGFs, CAFs had significantly increased proportion of pathologically activated fibroblasts. We next asked whether these CAFs were associated with GC progression. Transwell migration assay was performed and we found that compared with NGFs, CAFs promoted more cancer cells migrated through the membrane (Fig. 1C&D). Next we used NGFs and CAFs conditioned media to perform transwell assays. We found that CAFs-conditioned media also attracted more migrated cancer cells compared with NGFs-conditioned media (Fig. 1E&F). We then investigate the expression of MMP11 in CAFs and CAFs released exosomes. We identified that compared with NGFs and NGFs released exosomes, MMP11 was significantly increased in CAFs and CAFs released exosomes respectively, as shown in Fig. 1 G&H.

### Overexpression of MMP11 in exosomes of gastric CAFs contributed to gastric cancer cell migration

**Figure 2:**
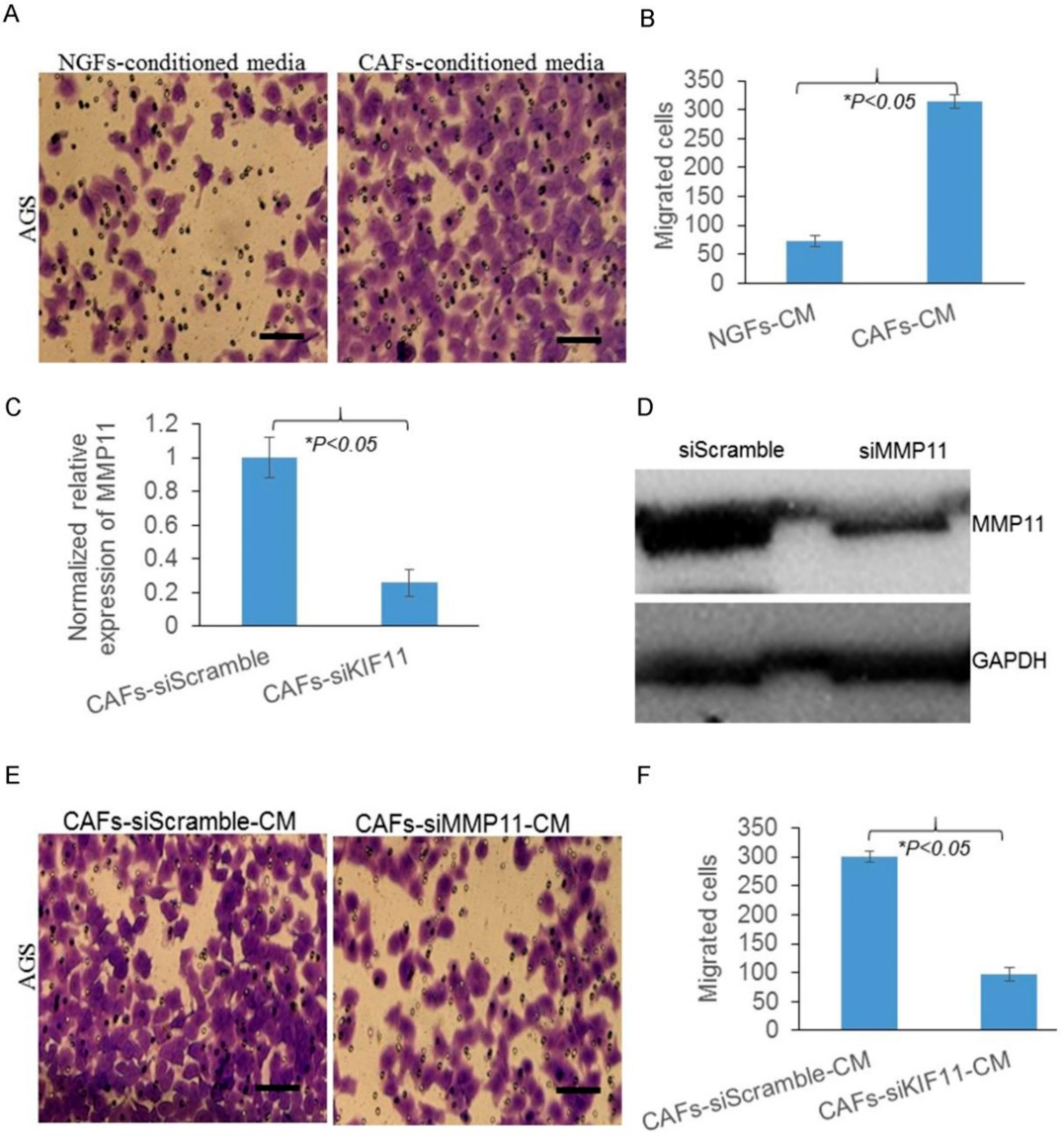
Gastric CAFs promotes gastric cancer cell migration partially through exosomal MMP11

As we know exosomes contain nucleic acids for intercellular communication. To investigate how CAFs promote gastric cancer cell migration, exosomes were successfully isolated (Fig. 2A). Moreover, CAFs had more exosomes released than NGFs (Fig. 2B). To study whether MMP11 was involved, we then investigated the expression of MMP11 in CAFs and CAFs released exosomes. We found that compared with NGFs and NGFs released exosomes, MMP11 was significantly increased in CAFs and CAFs released exosomes respectively as Fig. 2C&D showed.

Next, we used siRNA to knockdown the expression of MMP11, as Fig. 2 E&F showed, MMP11 expression was successfully suppressed by siRNA. And we found the numbers of migrated cells were significantly decreased in MMP11 knockdown group. All these results indicated that gastric CAFs promoted gastric cancer cell migration partially through exosomal MMP11.

### MMP11 was overexpressed in exosomes purified from plasma and tumor tissues of GC patients

**Figure 3:**
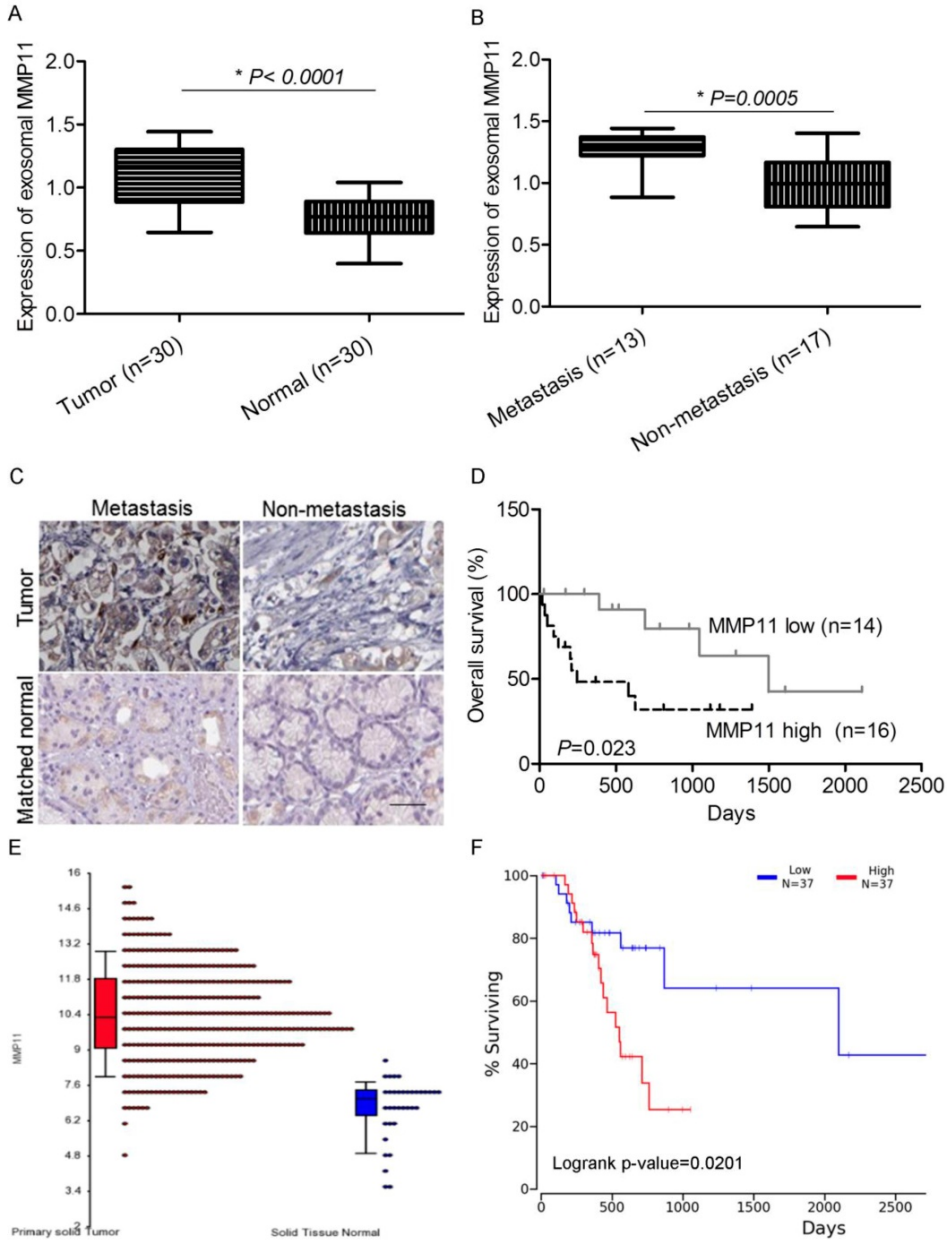
MMP11 is overexpressed in exosomes purified from plasma of gastric cancer patients and tumor tissues

MMP11 has been implicated to promote cancer development and progression in many malignancies. In our study, we analyzed its clinical significance as prognostic biomarker. In GC patients, we found that the expression of MMP11 in exosomes purified from plasma was significantly increased compared with healthy controls (Fig. 3A). Also, compared with non-metastasis GC patients, MMP11 was significantly increased in metastatic GC patients (Fig. 3B), indicating that MMP11 was related to GC and its progression and metastasis. We also detected the expression of MMP11 in GC tumor tissues and matched normal tissues, we found that MMP11 was significantly increased in GC tumors compared with matched nontumoral tissues, and also elevated in metastatic compared with non-metastatic tumors (Fig. 3C). Interestingly, we also found that the patients with low MMP11 expression had significantly better overall survival (Fig 3. D). Next we searched TCGA database, we found that MMP11 is overexpressed in GC tumors and is markedly associated with dismal prognosis (Fig 2. E&F).

### MMP11 was negatively regulated by exosomal miR-139 in the CAFs of gastric cancer

**Figure 4:**
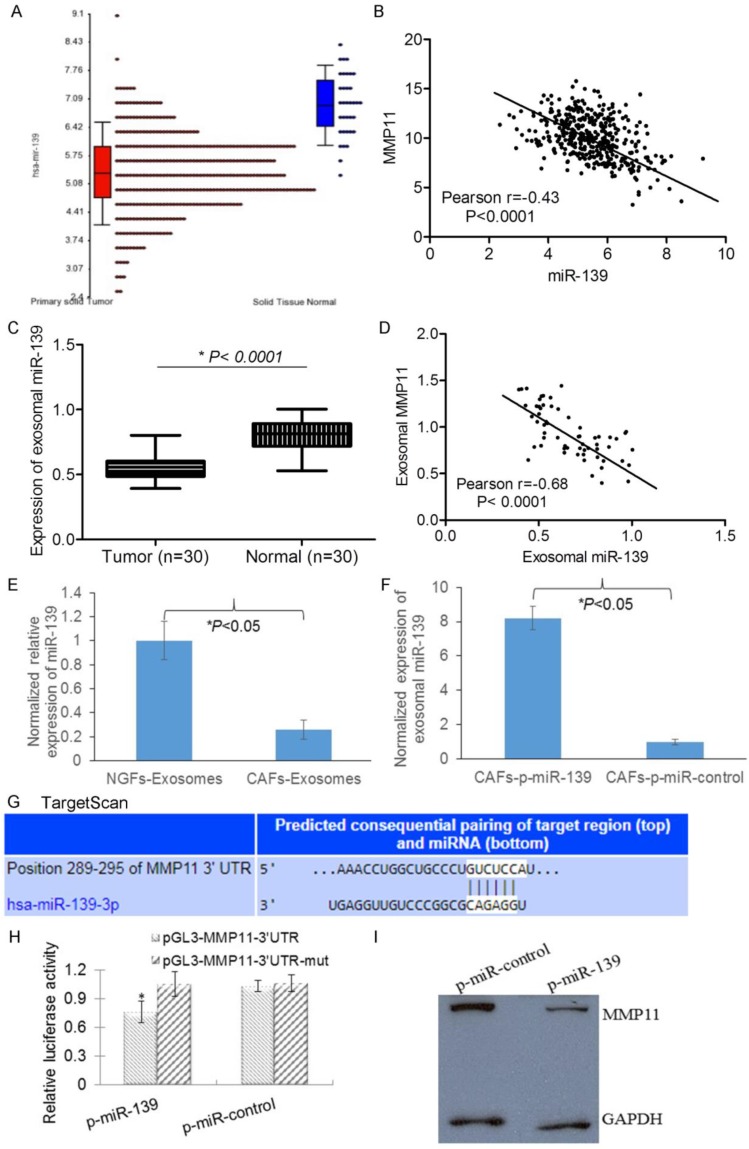
MMP11 is negatively regulated by exosomal miR-139 in the CAFs of gastric cancer

miRNAs have been implicated to regulate approximately 1/3 of all human genes. We next asked whether miRNA could regulate MMP11 in GC cells. We used Targetscan to predict that miR-139 may target MMP11. First we found that miR-139 decreased in GC tumors compared with matched nontumoral tissues in TCGA database. Meanwhile, the expression of MMP11 and miR-139 was inversely correlated in GC tumors (Fig.4 A&B), suggesting that miR-139 might regulate MMP11 in GC. We next analyzed the expression of miR-139 and MMP11 in our GC patients, and consistently, we found that miR-139 was decreased in tumors compared with matched nontumoral tissues. Interestingly miR-139 in exosomes released by GC tumors showed inverse correlation with MMP11 (Fig 4. C&D), suggesting exosomal miR-139 could regulate MMP11 in GC. Then, we detected the expression of exosomal miR-139 in CAFs. We found that compared with NGFs, the expression of miR-139 was significantly decreased in CAFs (Fig. 4E). We used miR-139 mimics to increase CAFs exosomal miR-139 (Fig. 4F). To examine whether the upregulation of MMP11 by miR-139 is caused by direct binding to the putative targeting site in the MMP11, we constructed both wild and mutant luciferase reporter plasmid pMIR-MMP11-3′UTR containing the putative miR-139 binding site of MMP11 3′UTR downstream of the luciferase open reading frame. Luciferase report system showed a significant decrease in luciferase activity in pMIR-wt MMP11-3′UTR in the presence of miR-139 mimics, while no significant change in pMIR-mut MMP11-3′UTR (Fig 4.G&H). And more importantly, the expression of MMP11 was decreased in miR-139 mimics harbouring cells when compared with the control cells (Fig. 4I). These data suggest that miR-139 binds to the 3′ UTR of MMP11 and impairs MMP11 mRNA translationin CAFs.

### Exosomal miR-139 was transferred from CAFs to gastric cancer cells and inhibits cell growth and migration

**Figure 5:**
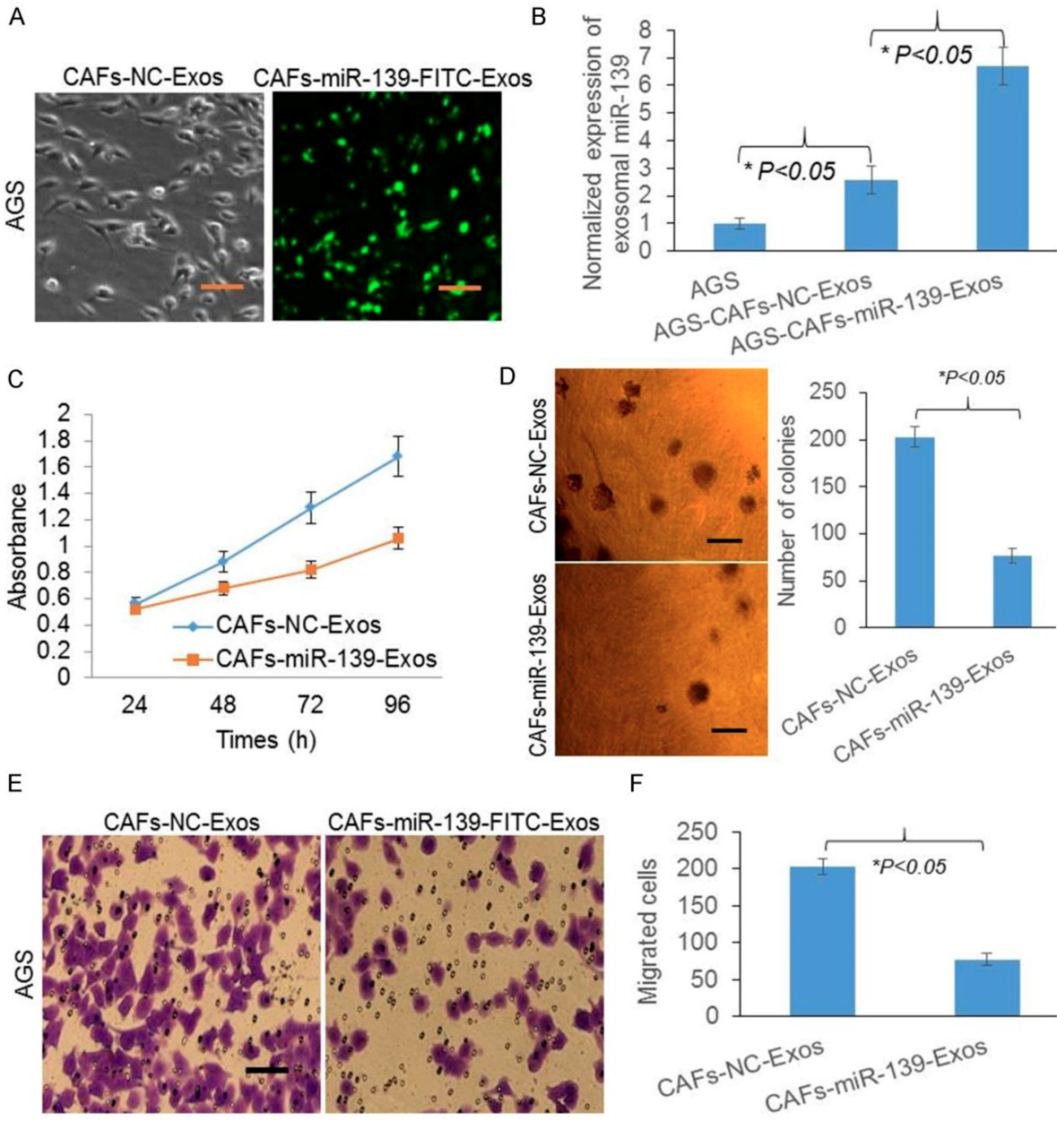
Exosomal miR-139 was transferred from CAFs to gastric cancer cells and inhibits cell growth and migration

Since miR-139 was marked with FITC, green fluorescence represented miR-139 in AGS (Fig. 5A). miR-139 levels were significantly increased after CAFs-miR-139-Exos treated (Fig. 5B), indicating exosomal miR-139 could be transferred from CAFs to gastric cancer cells. Next, to investigate the role of miR-139 in AGS, we detected the cell proliferation and colony formation of AGS. And we found both the absorbance (Fig. 5C) and number of colonies were decreased after miR-139 was transferred to AGS (Fig. 5D), suggesting miR-139 inhibited cell growth of cancer cells. Transwell migration assay was also performed and we found that miR-139 inhibited cancer cells migration in AGS (Fig. 5E&F).

### Exosomal miR-139 inhibited tumor growth and metastasis of gastric cancer cells through downregulating MMP11* in vivo*

**Figure 6:**
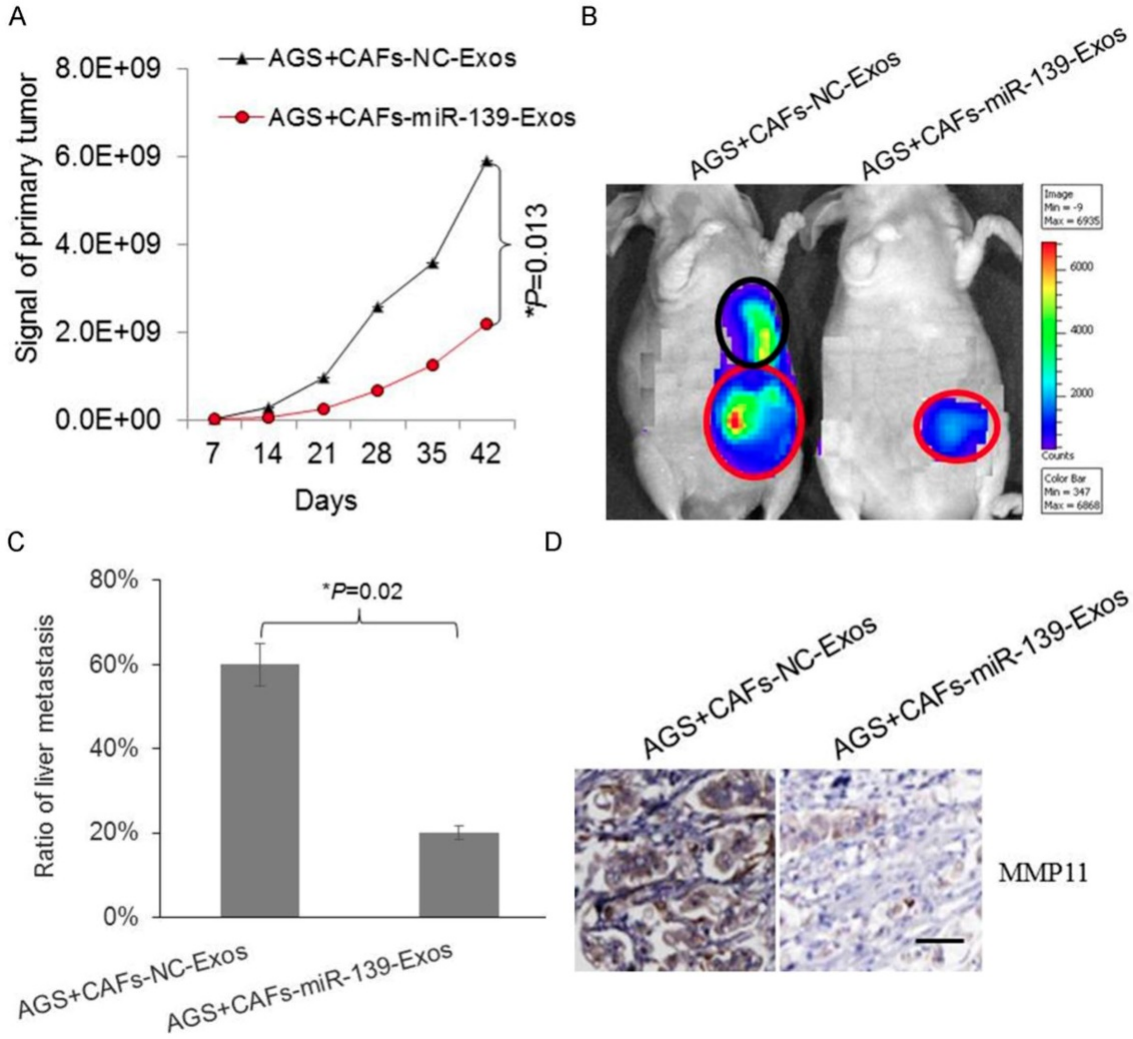
Exosomal miR-139 inhibits tumor growth and metastasis of gastric cancer cells with decreasing the expression of MMP11 *in vivo*

To further confirm the protective role of exosomal miR-139 in gastric cancer, AGS and miR-139-exosome cells were injected in mice. Bioluminescent imaging showed the signal of gastric primary tumor was lower in miR-139-exosome group (Fig. 6A). The metastasis of tumor primary site or metastasis site was observed by bioluminescent imaging (Fig. 6B). We analyzed the metastasis in liver according to the signal reflected by imaging. As Fig. 6C showed, ratio of liver metastasis was significantly lower in miR-139-exosome group, indicating exosomal miR-139 inhibited metastasis of gastric cancer cells. The expression of MMP11 in GC tumor tissues was also detected, and we found that MMP11 was significantly decreased in miR-139-exosome group compared with NC-exosome group (Fig. 6D); suggesting MMP11 was involved in the inhibitory effects of exosomal miR-139 on tumor growth and metastasis of GC cells.

## Discussion

CAFs have been found to play a key role by secreting various soluble factors to support tumor growth 1,2. However, the mechanisms underlying CAF's promotion of tumor metastasis remain largely unknown in GC. Exosomal miRNAs mediate cell.

To cell communication and play central roles in the crosstalk between cancer cells and the TME 9, 10. In the present study, we identified as a critical factor regulating cell migration and invasion. We showed that exosomal miR-139 was downregulated in CAFs. Through the regulation of MMP11 expression, suppression of exosomal miR-139 promoted the pro-tumor activity of CAFs in GC.

As one of the important stromal cells in solid tumors, CAFs differentiate into fibroblasts that express α-SMA, playing a central role in promoting tumor growth and progression 17. Many studies have shown that the accumulation of CAFs in tumor stroma accelerates metastasis 5,18-20. High expression of α-SMA was found both in GC tumors and CAFs, indicating a pathogenic role of fibroblasts in GC tumor. Since CAFs conditioned media attracted more migrated cancer cells, there might be cytokines, chemokines or other factors contributing to invasion and metastasis of GC cells. Exosome can be taken up by neighboring or distant cells, subsequently leading to changes in gene expression, and it plays a crucial role in cancer biology with vesicular transport 21-26. In our study, exosomal MMP11 was found overexpressed in gastric CAFs. MMPs are zinc-dependent endopeptidases, which are key regulators of extracellular matrix degradation. The overexpression of many MMP family members, such as MMP2, MMP9 and MMP11 has been found to be involved in cancer progression 27-29. In this study, we found MMP11 in exosomes of CAFs promoted cancer cell migration. This can be a new mechanism explained how CAFs communicate with cancer cells to promote GC. Overexpressed MMP11 in plasma exosomes was highly associated with inferior survival rate of GC patients, which can be a prognostic indicator of GC.

miRNAs, whose dysregulation has been verified in several types of cancers 30, can be stably transferred by exosome 31. Except for serum, exosomal miRNAs have also been described in saliva and urine. In this study, exosomal miR-139 was found in CAFs. Also, we found exosomal miR-139 associated with the interactions between CAFs and cancer cells. When exosomal miR-139 was transferred from CAFs to gastric cancer cells, both cell growth and migration were inhibited. Recent studies revealed miRNA dysregulation plays an important role in controlling the secretory function of CAFs 32-34. By altering the secretory phenotype of cancer cells, the communication between CAFs and cancer cells can be affected. Consistent with it, we found exosomal miR-139 downregulated MMP11 in CAFs, inhibiting cancer cells migration, and further inhibited metastasis of GC cells.

In summary, we have demonstrated that stromal MMP11 overexpression predicted poor survival in human GC. Furthermore, exosomal miR-139 was under-expressed in CAFs, and promoted the expression of MMP11, resulting in increased growth, invasion and metastasis of GC cells in vitro and in vivo. Our findings reveal the regulation of MMP11 by exosomal miR-139 in CAFs associated with gastric cancer progression. Investigating how this signal pathway influenced tumor cells will improve our understanding of the mechanisms underlying GC progression, and help develop new targets for its therapy.

## References

[1] Franco OE, Shaw AK, Strand DW (2010). Cancer associated fibroblasts in cancer pathogenesis. Semin Cell Dev Biol.

[2] Chen W, Zheng R, Baade PD (2016). Cancer statistics in China, 2015. CA Cancer J Clin.

[3] Cirri P, Chiarugi P (2011). Cancer associated fibroblasts: the dark side of the coin. Am J Cancer Res.

[4] Ma Z, Chen M, Yang X (2018). The Role of Cancer-associated Fibroblasts in Tumorigenesis of Gastric Cancer. Curr Pharm Des.

[5] Yan Y, Wang LF, Wang RF (2015). Role of cancer-associated fibroblasts in invasion and metastasis of gastric cancer. World J Gastroenterol.

[6] Ma Y, Zhu J, Chen S (2019). Low expression of SPARC in gastric cancer-associated fibroblasts leads to stemness transformation and 5-fluorouracil resistance in gastric cancer. Cancer Cell Int.

[7] EL Andaloussi S, Mäger I, Breakefield XO (2013). Extracellular vesicles: biology and emerging therapeutic opportunities. Nat Rev Drug Discov.

[8] Thery C, Zitvogel L, Amigorena S (2002). Exosomes: composition, biogenesis and function. Nat Rev Immunol.

[9] Hu JL, Wang W, Lan XL (2019). CAFs secreted exosomes promote metastasis and chemotherapy resistance by enhancing cell stemness and epithelial-mesenchymal transition in colorectal cancer. Mol Cancer.

[10] Hu HY, Yu CH, Zhang HH (2019). Exosomal miR-1229 derived from colorectal cancer cells promotes angiogenesis by targeting HIPK2. Int J Biol Macromol.

[11] Yang H, Fu H, Wang B (2018). Exosomal miR-423-5p targets SUFU to promote cancer growth and metastasis and serves as a novel marker for gastric cancer. Mol Carcinog.

[12] Yoshii S, Hayashi Y, Iijima H (2019). Exosomal miRNAs derived from colon cancer cells promote tumor progression by suppressing fibroblast TP53 expression. Cancer Sci.

[13] Lee YR, Kim G, Tak WY (2019). Circulating exosomal noncoding RNAs as prognostic biomarkers in human hepatocellular carcinoma. Int J Cancer.

[14] Kim S, Choi MC, Jeong JY (2019). Serum exosomal miRNA-145 and miRNA-200c as promising biomarkers for preoperative diagnosis of ovarian carcinomas. J Cancer.

[15] Marsh D, Suchak K, Moutasim KA (2011). Stromal features are predictive of disease mortality in oral cancer patients. J Pathol.

[16] Livak KJ, Schmittgen TD (2001). Analysis of relative gene expression data using real-time quantitative PCR and the 2(-Delta Delta C(T)) method. Methods.

[17] Herrera M, Islam AB, Herrera A (2013). Functional heterogeneity of cancer associated fibroblasts from human colon tumors shows specific prognostic gene expression signature. Clin Cancer Res.

[18] Zhi K, Shen X, Zhang H (2010). Cancer-associated fibroblasts are positively correlated with metastatic potential of human gastric cancers. J Exp Clin Cancer Res.

[19] Erdogan B, Webb DJ (2017). Cancer-associated fibroblasts modulate growth factor signaling and extracellular matrix remodeling to regulate tumor metastasis. Biochem Soc Trans.

[20] Kashima H, Noma K, Ohara T (2019). Cancer-associated fibroblasts (CAFs) promote the lymph node metastasis of esophageal squamous cell carcinoma. Int J Cancer.

[21] Kogure A, Kosaka N, Ochiya T (2019). Cross-talk between cancer cells and their neighbors via miRNA in extracellular vesicles: an emerging player in cancer metastasis. J Biomed Sci.

[22] Bronisz A, Godlewski J, Chiocca EA (2016). Extracellular vesicles and MicroRNAs: their role in Tumorigenicity and therapy for brain tumors. Cell Mol Neurobiol.

[23] Naito Y, Yamamoto Y, Sakamoto N (2019). Cancer extracellular vesicles contribute to stromal heterogeneity by inducing chemokines in cancer-associated fibroblasts. Oncogene.

[24] Zeng Z, Li Y, Pan Y (2018). Cancer-derived exosomal miR-25-3p promotes pre-metastatic niche formation by inducing vascular permeability and angiogenesis. Nat Commun.

[25] Campos A, Salomon C, Bustos R (2018). Caveolin-1-containing extracellular vesicles transport adhesion proteins and promote malignancy in breast cancer cell lines. Nanomedicine (Lond).

[26] Huang Z, Yang M, Li Y (2018). Exosomes Derived from Hypoxic Colorectal Cancer Cells Transfer Wnt4 to Normoxic Cells to Elicit a Prometastatic Phenotype. Int J Biol Sci.

[27] Peruzzi D, Mori F, Conforti A (2009). MMP11: a novel target antigen for cancer immunotherapy. Clin Cancer Res.

[28] Perigny M, Bairati I, Harvey I (2008). Role of lmmunohistochemical overexpression of matrix metalloproteinases MMP-2 and MMP-11 in the prognosis of death by ovarian cancer. Am J Clin Pathol.

[29] Wang B, Hsu CJ, Lee HL (2018). Impact of matrix metalloproteinase-11 gene polymorphisms upon the development and progression of hepatocellular carcinoma. Int J Med Sci.

[30] Halvorsen AR, Helland A, Gromov P (2017). Profiling of microRNAs in tumor interstitial fluid of breast tumors - a novel resource to identify biomarkers for prognostic classification and detection of cancer. Mol Oncol.

[31] Ge Q, Zhou Y, Lu J (2014). miRNA in plasma exosome is stable under different storage conditions. Molecules.

[32] Schoepp M, Ströse AJ, Haier J (2017). Dysregulation of miRNA Expression in Cancer Associated Fibroblasts (CAFs) and Its Consequences on the Tumor Microenvironment.

[33] Yang F, Ning Z, Ma L (2017). Exosomal miRNAs and miRNA dysregulation in cancer-associated fibroblasts. Mol Cancer.

[34] Hook LM, Grey F, Grabski R (2014). Cytomegalovirus miRNAs target secretory pathway genes to facilitate formation of the virion assembly compartment and reduce cytokine secretion. Cell Host Microbe.

